# Supplementary material for: Iterative improvement in the automatic modular design of robot swarms
Source: PeerJ Comput Sci. 2020 Dec 7;6:e322. doi: 10.7717/peerj-cs.322 (PMC7924708; doi:10.7717/peerj-cs.322)
Supplement: Supplemental Information 3 [file peerj-cs-06-322-s003.zip › argos3/doc/api/standalone/a00561_source.html]

ARGoS: plugins/robots/spiri/simulator/qtopengl\_spiri.h Source File


- Main Page
- Related Pages
- Namespaces
- Classes
- Files

- File List
- File Members

# plugins/robots/spiri/simulator/qtopengl\_spiri.h

Go to the documentation of this file.

```
00001 
00007 #ifndef QTOPENGL_SPIRI_H
00008 #define QTOPENGL_SPIRI_H
00009 
00010 namespace argos {
00011    class CQTOpenGLSpiri;
00012    class CSpiriEntity;
00013 }
00014 
00015 
00016 #ifdef __APPLE__
00017 #include <gl.h>
00018 #else
00019 #include <GL/gl.h>
00020 #endif
00021 
00022 namespace argos {
00023 
00024    class CQTOpenGLSpiri {
00025 
00026    public:
00027 
00028       CQTOpenGLSpiri();
00029 
00030       virtual ~CQTOpenGLSpiri();
00031 
00032       virtual void Draw(CSpiriEntity& c_entity);
00033 
00034    private:
00035 
00036       void SetPlasticMaterial();
00037 
00038       void SetPropellerMaterial();
00039 
00040       void SetMainBodyMaterial();
00041 
00042       void MakeModel();
00043 
00044       void MakePropeller();
00045 
00046       void MakeMainBody();
00047 
00048       void MakeRing(GLfloat f_out_radius,
00049                     GLfloat f_in_radius);
00050 
00051       void MakeDisk(GLfloat f_radius);
00052 
00053       void MakeCylinderSurface(GLfloat f_radius,
00054                                GLfloat f_height);
00055 
00056       void MakeCylinder(GLfloat f_radius,
00057                         GLfloat f_height);
00058 
00059       void MakeBox(GLfloat f_length,
00060                    GLfloat f_width,
00061                    GLfloat f_height);
00062 
00063    private:
00064 
00065       /* Display list */
00066       GLuint m_unList;
00067 
00068       /* Number of vertices to display the round parts */
00069       GLuint m_unVertices;
00070 
00071    };
00072 
00073 }
00074 
00075 #endif
```

---

Generated on 10 Jul 2018 for ARGoS by 
 1.6.1
